# Acute radiation syndrome caused by accidental radiation exposure - therapeutic principles

**DOI:** 10.1186/1741-7015-9-126

**Published:** 2011-11-25

**Authors:** Harald Dörr, Viktor Meineke

**Affiliations:** 1 Bundeswehr Institute of Radiobiology, affiliated to the University of Ulm, Neuherbergstr. 11, 80937 Munich, Germany

## Abstract

Fortunately radiation accidents are infrequent occurrences, but since they have the potential of large scale events like the nuclear accidents of Chernobyl and Fukushima, preparatory planning of the medical management of radiation accident victims is very important. Radiation accidents can result in different types of radiation exposure for which the diagnostic and therapeutic measures, as well as the outcomes, differ. The clinical course of acute radiation syndrome depends on the absorbed radiation dose and its distribution. Multi-organ-involvement and multi-organ-failure need be taken into account. The most vulnerable organ system to radiation exposure is the hematopoietic system. In addition to hematopoietic syndrome, radiation induced damage to the skin plays an important role in diagnostics and the treatment of radiation accident victims. The most important therapeutic principles with special reference to hematopoietic syndrome and cutaneous radiation syndrome are reviewed.

## Introduction

Fortunately, radiation accidents are infrequent occurrences, but since they have the potential of resulting in large scale events, such as the nuclear accidents of Chernobyl and Fukushima, preparatory planning for medical management of radiation accident victims is very important [1].

Radiation accidents can result in radiation exposure to only a few up to several hundreds of people, depending on the type of accident and the amount of radiation exposure. Radiation exposure can occur as external exposure, for instance, from a sealed radiation source, or as internal exposure due to the intake of radionuclides. In a nuclear disaster scenario like the Chernobyl accident, a combination of external and internal radiation exposure could occur. Radiation exposure can also be categorized as either chronic or acute, depending on the period of time of radiation exposure. Another important factor that can effect treatment and outcome is whether the whole body of a person was affected homogeneously or if only localized radiation exposure of a part of the body occurred.

For each of these different types of scenarios and combinations the expected consequences for the patient, depending on the absorbed radiation dose and distribution as well as diagnostic and therapeutic measures, are different. In the case of whole body exposure, all organs and organ systems are affected and, therefore, multi-organ-involvement and multi-organ-failure have always to be taken into account. Additional conventional trauma, such as wounds and burns in people with severe radiation exposure - the so-called radiation combined injury, could worsen the prognosis [2].

There are many aspects to consider when diagnosing and managing radiation exposed patients. Acute versus chronic effects can be differentiated by the latency of manifestation of the radiation effects. Since acute effects require immediate therapeutic intervention, they should be diagnosed at an early stage. Another differentiation could be made between deterministic versus stochastic effects regarding their pathophysiological mechanisms. Deterministic radiation effects, such as the hematopoietic syndrome, occur after a threshold radiation dose is exceeded and the severity will increase with increasing radiation exposure. For stochastic effects like the development of malignant tumors on the other hand, the probability of their appearance will increase with increasing radiation exposure.

Therefore, all efforts must be made to reduce the individual exposure to ionizing radiation and, thus, the absorbed dose [3].

The identification of external contamination with radionuclides is important, since in the case of external contamination patients have to be decontaminated as soon as possible and special precautions must be taken to protect first responders, medical personnel, facilities and equipment from contamination. If patients have incorporated radionuclides, a specific de-corporation therapy must be carried out as soon as possible to reduce the resulting radiation dose. Since the de-corporation therapy has to be specific for the involved radionuclide, the identification of the radionuclide must be the initial step [1]. Even though guidelines for de-corporation therapy are available on national and international levels, some of the substances are not approved for this purpose in all countries and "off label use" might be required [4].

For the planning of the medical management of radiation accident victims, it is crucial to estimate the severity of the radiation exposure on the basis of the individual patient's clinical signs and symptoms by means of a clinical dosimetry.

After significant acute whole body or partial body radiation exposure, resulting in acute radiation induced health impairments, it is imperative that appropriate therapeutic measures be carried out as soon as possible.

When dealing with specific recommendations for countermeasures against radiation-induced health impairments, the main fact is that due to the comparatively low number of radiation victims worldwide, there is a clear lack of controlled studies in this area. Therefore, established and accepted animal models [5], as well as recommendations of national and international expert panels and committees in this field [6-8], are the main sources of information. Still there remains uncertainty in many areas, which is the basis for future research. This review, therefore, will mainly focus on established therapeutic measures in the clinical management of radiation accident victims suffering from acute radiation effects.

## Acute radiation syndrome (ARS)

Depending on the magnitude of the radiation exposure resulting in significant whole body exposure or partial body exposure, the patient will develop clinically acute radiation effects resulting in acute radiation syndrome (ARS).

The biological effects of ionizing radiation will start on the cellular level by energy absorption due to several physical effects, such as the Compton process and the photoelectric process for x- or gamma-rays. The most important targets are the DNA-molecules, where direct or indirect actions of radiation could result in lesions, such as base damage, single-strand breaks and double-strand breaks. Double-strand breaks are considered the most serious DNA-lesions, since they can result in the cleavage of chromatin and might not be successfully repaired by the cell. The occurrence of DNA-lesions and, especially, of double-strand breaks will increase with increasing radiation exposure and will lead to a higher risk of cell death [3].

The classical initial symptoms of acute radiation syndrome occur during the so-called prodromal phase. Prodromal symptoms include: anorexia, nausea, vomiting, diarrhoea, fluid loss, fever, hypotension, headache and early erythema [3,9]. These prodromal symptoms could, if the possibility of a radiation exposure is not taken into account, be misinterpreted as unspecific symptoms of gastrointestinal or other infectious diseases. For this reason, the possibility of radiation-induced health impairments should always be taken into account, if unspecific symptoms cannot be properly explained. The prodromal phase is followed by the latent phase. In the latent phase, symptoms will decrease or even disappear. The length of the latent phase depends on the magnitude of the radiation exposure. After a very high radiation exposure it can also be missing. The latent phase will be followed by the manifestation phase. The occurrence and severity of clinical signs and symptoms will depend on the absorbed radiation dose. Depending on the absorbed radiation dose, the manifestation takes place in different organ systems as syndromes of the hematopoietic system, the gastrointestinal system, the skin and the neurovascular system. Hematopoietic syndrome will occur at a lower dose than the other syndromes due to the high radiosensitivity of the hematopoietic system. Even in asymptomatic patients effects on the blood cell counts might be observed. A complete radiation-induced failure of the hematopoietic system on the other hand requires an ample homogenous whole body exposure for all hematopoietic stem cells in the bone marrow to be irreversibly damaged.

With higher radiation exposure, disturbances of the gastrointestinal system, such as destruction of the mucosal layer can take place. A complete loss of the mucosal layer will be fatal. Very high radiation exposure can result in neurologic and cardiovascular breakdown causing death within a few days. The cutaneous syndrome can occur together with the other syndromes, but cutaneous radiation injury (CRI) could also be the consequence of external exposure to beta-radiation in the absence of other symptoms of ARS [3,9].

All differentiated cells and stem cell pools of the organism will be affected from acute homogeneous whole body radiation exposure resulting in multi-organ involvement (MOI) and even multi-organ failure (MOF).

Pathophysiological aspects of radiation-induced MOI include systemic inflammatory response syndrome (SIRS) and consequences of cell loss due to radiation damage [10]. Therapeutic efforts are to be taken to stabilize the homoeostasis and to reconstitute the function of organs and organ systems. A new strategy would be an early therapeutic intervention in order to prevent MOF already in the stage of SIRS. The pathophysiological mechanisms behind this development are still poorly understood [11].

## Therapeutic principles in clinical management of patients with ARS

Clinical management of patients with acute radiation syndrome is characterized by dealing with radiation induced impairments of different organ functions, multi-organ involvement or even multi-organ failure.

In the early stages of the accident situation, reliable information about physical dosimetry and results from biodosimetric methods are not always immediately available. Therefore, the estimation of radiation effects and the patient's prognosis will be based on clinical signs and symptoms as described in the METREPOL system [12]. Instead of making therapeutic decisions only based on information about the absorbed radiation dose, the patient's clinical status will be categorized into response categories (RC) 1 to 4. According to the METREPOL system, organ specific check lists will be used for the grading of radiation effects in the four most important organ systems, such as the neurovascular system (N), the hematopoietic system (H), the cutaneous system (C) and the gastrointestinal system (G). Different levels of the severity of organ system specific clinical signs and symptoms will then result in response categories, for example, from H1 to H4 for the hematopoietic system. The organ specific grading will then lead to a resulting RC for the individual patient. These response categories describe the degree of radiation-induced damage but also include prognostic aspects. The definition of the four response categories are as follows: RC 1 for mild damage, RC 2 for moderate damage, RC 3 for severe damage and RC 4 for serious or fatal damage [12].

As soon as reliable information about the physical dose or results from biodosimetry is available, the data should be included in therapeutic decision making and sufficient medical management. It is the prediction of expected radiation-induced impairments of organs or organ-systems that is important for this management.

One of the most critical and most vulnerable organ systems to radiation exposure is the hematopoietic system, since the limited life-span of blood cells requires continuous cell divisions of hematopoietic stem cells in the bone marrow. The impairment of the hematopoiesis will result in pancytopenia of various degrees with consecutive increased risk of infection, hemorrhage and anemia. General medical management consists of barrier nursing conditions, sufficient and immediate therapy of infections or even prophylactic administration of antibiotic, and antimycotic and antiviral substances [12-14]. Since renal function is of great importance for maintaining homoeostasis, findings concerning the effectiveness of angiotensin-converting enzyme inhibitors and angiotensin II type 1 receptor antagonists in reducing the incidence and severity of chronic renal and lung injuries have to be taken into account [15-17]. A preclinical experimental study regarding the therapeutic intervention in the gastrointestinal tract showed promising results. Administration of the somatostatin analog SOM230 significantly increased the survival rate when started 48 hours after radiation exposure [18]. Other possible radiation countermeasures include cytokines, growth factors and antioxidants which are able to scavenge free radicals and modulate cell death signaling or cell cycle progression [19].

Depending on the severity of the hematopoietic syndrome, the main therapeutic principles are replacement with blood products, such as erythrocyte concentrate, the administration of cytokines like granulocyte colony-stimulating factor (G-CSF) and granulocyte-macrophage colony-stimulating factor (GM-CSF), and the transplantation of hematopoetic stem cells (HSCT) [20]. The source of hematopoietic stem cells for HSCT can be bone marrow, mobilized peripheral blood-derived stem cells, umbilical cord blood or the fetal liver [21].

The therapeutic use of hematopoietic factors such as G-CSF, GM-CSF, erythropoietin (EPO) and thrombopoietin (TPO) has been described in several cases [22-24]. Since the number of radiation-exposed patients treated with hematopoietic factors is limited and randomized controlled clinical trials cannot be performed after radiation accidents, the main supporting evidence for the effectiveness of hematopoietic factors in ARS is based on experimental animal studies [20,25,26]. Since experimental animal studies are of such great importance in the field of radiation effects, they have to meet certain standards to allow a comparison of the results [5].

The therapeutic use of hematopoietic factors in radiation accident victims will be considered as "off-label use". But if the development of severe neutropenia in a patient is expected, the administration of G-CSF or GM-CSF in an early stage is recommended [6,8,14].

If unrecoverable damage to the hematopoietic stem cell pool is noticed, a decision about the necessity of HSCT has to be made. The diagnosis, whether or not an autologous recovery of the hematopoiesis could be expected, requires specific expertise [7,21,27,28]. The experience from the treatment of patients after several radiation accidents with HSCT showed that the range between the beneficial treatment with HSCT and a very poor prognosis irrespective of whether HSCT is performed or not, is quite narrow, especially if other organ systems are severely affected or MOI already occurred [21,29,30]. HSCT will, therefore, not be considered as the most important treatment option in ARS, although it could be essential for an individual patient.

In addition to hematopoetic syndrome, radiation-induced damage to the skin plays an important role in diagnosis and treatment of patients with ARS, but also in the case of local radiation injuries. The impairment of the skin could be a real challenge in the clinical management of patients with cutaneous radiation syndrome. The barrier function of the skin will be affected and inflammatory reactions will take place, which eventually might trigger the development of MOF [31,32]. Therapeutic principles in the clinical management of patients with cutaneous radiation syndrome include conservative methods, surgical treatment and the administration of anti-inflammatory agents and topical steroids [8]. Systemic administration of steroids could be considered for MOF-related skin dysfunction [23,33]. A novel therapeutic approach is the parenteral or local administration of mesenchymal stem cells [34]. The treatment of a patient with a local radiation injury using local cellular therapy with autologous expanded mesenchymal stem cells to promote tissue regeneration resulted in favorable pain relief and healing progression [35]. As conservative methods, therapeutic measures for pain control, reduction of inflammation, prevention of infection and of further vasculature insult, improvement of circulation, healing acceleration, wound cleaning and minimizing fibrosis will be performed. Surgical treatment and skin grafts might be required if necrosis of various extents occur [32,36]. In order to avoid disturbances of wound healing after exposure to ionizing radiation, all surgical measures within the ARS should be performed as soon as possible [37]. New approaches, such as mesenchymal stem cell administration derived from experimental studies in animal models, should be considered in patients with cutaneous radiation syndrome [38-42].

## Summary and future directions

The development of acute radiation effects in radiation accident victims depends on the nature and the extent of radiation exposure. Since reliable information about the radiation exposure from physical dosimetry and results from biodosimetric methods are usually not available in accident situations, the estimation of radiation effects can be performed on the basis of clinical signs and symptoms as described in the METREPOL system.

Multi-organ involvement, systemic inflammatory response syndrome and even multi-organ failure have to be considered in the clinical management of radiation accident victims.

Since the hematopoietic system is most vulnerable to ionizing radiation, diagnostic and therapeutic measures dealing with the hematopoietic syndrome are most important. General measures are barrier nursing conditions, sufficient and immediate therapy of infections, or even prophylactic administration of antibiotic, antimycotic and antiviral substances. The main specific therapeutic principles are replacement with blood products, the administration of cytokines like G-CSF and GM-CSF, and the transplantation of hematopoietic stem cells.

In addition to hematopoietic syndrome, radiation-induced damage to the skin plays an important role in diagnostics and treatment of patients with ARS and eventually might trigger the development of multi-organ failure.

New approaches are based on the administration of stem cells, such as mesenchymal stem cells, in the case of cutaneous radiation syndrome and localized radiation injuries. Since radiation-induced multi-organ involvement or multi-organ failure will be associated with a poor prognosis in the patient, early therapeutic intervention for preventing the development of MOI and MOF seems to be one of the most important aspects for further research.

## Conclusions

From past experiences, we know that radiation accidents fortunately are rare events; therefore, the number of patients suffering from acute radiation effects and ARS is limited. However, current risk analyses of terrorist threats consider a nuclear scenario as extremely relevant. This certainly would mean that the number of victims would be in a much higher magnitude. Availability of necessary resources could be a limiting factor for the medical management of radiation accident victims. Stockpiling of essential pharmaceuticals for the treatment of radiation accident victims on a national or international level has to be considered.

Prospective studies for the development of therapeutic standards for patients with ARS are extremely limited. Most of the new approaches in therapeutic measures are derived from experimental studies in animal models. Keeping these circumstances in mind, a multidisciplinary approach built on international cooperation is of the utmost importance and currently the most reasonable strategy to provide the best possible medical care for radiation accident victims. The clinical course, as well as the therapeutic regimen, of each new radiation accident victim should be documented in detail for further analysis, as in the SEARCH database [43].

There is a strong need for internationally recognized guidelines for the treatment of severely radiation-exposed patients [8]. Further research and experimental studies are necessary to identify prognostic parameters for the estimation of irreversible damage to organs and organ systems and a deeper understanding of the pathophysiology of radiation induced MOF.

## Abbreviations

ARS: acute radiation syndrome; CRI: cutaneous radiation injury; CRS: cutaneous radiation syndrome; G-CSF: granulocyte colony-stimulating factor; GM-CSF: granulocyte-macrophage colony-stimulating factor; Gy: Gray; HSCT: hematopoetic stem cell transplantation; M-CSF: macrophage - colony-stimulating factor; METREPOL: Medical Treatment Protocols for Radiation Accident; MOI: multi-organ involvement; MOF: multi-organ failure; MSC: mesenchymal stem cells; RC: response category; Sv: Sievert; SIRS: systemic inflammatory response syndrome.

## Competing interests

The authors declare that they have no competing interests.

## Authors' contributions

HD and VM have made substantial contributions, drafted and revised the manuscript and both have given final approval of the version to be published.

## Pre-publication history

The pre-publication history for this paper can be accessed here:

http://www.biomedcentral.com/1741-7015/9/126/prepub
